# Prevalence and regional disparities in probable mental health conditions among persons aged 10 years and older in Uganda: Evidence from the 2024 National Population and Housing Census

**DOI:** 10.1017/gmh.2026.10237

**Published:** 2026-05-28

**Authors:** Herbert Elvis Ainamani, Joseph Namanya, Nolbert Gumusiriza, Herbert Izo Ninsiima, Sarah M. Arinaitwe, Ronald Bahati, Godfrey Zari Rukundo

**Affiliations:** 1 Department of Psychiatry, Kabale University School of Medicine, Uganda; 2 Department of Statistical Methods and Actuarial Science, Makerere University College of Humanities and Social Sciences, Uganda; 3 Department of Human Pysiology, Kabale University School of Medicine, Uganda; 4 Department of Child and Child care and development, HOPE Partners-Africa, Uganda; 5 Department of Psychology, Kyambogo University, Uganda; 6 Department of Psychiatry and Neurobehavioral Sciences, McMaster University, Canada; 7 Department of Psychiatry, Mbarara University of Science and Technology Faculty of Medicine, Uganda

**Keywords:** mental health conditions, psychological distress, population census, Uganda, *sociodemographic disparities*

## Abstract

Despite the growing global burden of mental health conditions, nationally representative data in low- and middle-income countries remain limited. Using data from the 2024 Uganda National Population and Housing Census, this study examined the prevalence and distribution of probable mental health conditions among persons aged 10 years and older in Uganda, while assessing variations by sex, place of residence and sub-region. We conducted a descriptive secondary analysis of publicly available census tabulations (2024 Volume I, Section 6.5). The analytic population comprised 31,389,721 individuals aged 10 years and older. Mental health outcomes were based on self-reported symptoms synthesised by the Uganda Bureau of Statistics into six indicators of probable mental health conditions, such as general psychological distress, depression, anxiety, bipolar disorder, psychosis and suicidality, rather than clinical diagnoses. Among persons aged 10 years and older, 3,773,915 (12.0%) reported probable general psychological distress. Probable depressive condition affected 2,444,272 (7.8%), followed by probable bipolar condition 1,262,088 (4.0%) and probable anxiety condition 1,201,182 (3.8%). Higher prevalence was observed among females and rural residents. Substantial sub-regional variation was observed across all outcomes, with the prevalence of general psychological distress ranging from ~8% in Madi, Sebei and Ankole to over 16% in Teso. The 2024 census suggests a substantial and uneven distribution of probable mental health conditions in Uganda and underscores the need for geographically targeted, equity-oriented mental health strategies, as well as stronger integration of mental health into primary healthcare systems.

## Graphical abstract

**Figure 1:**
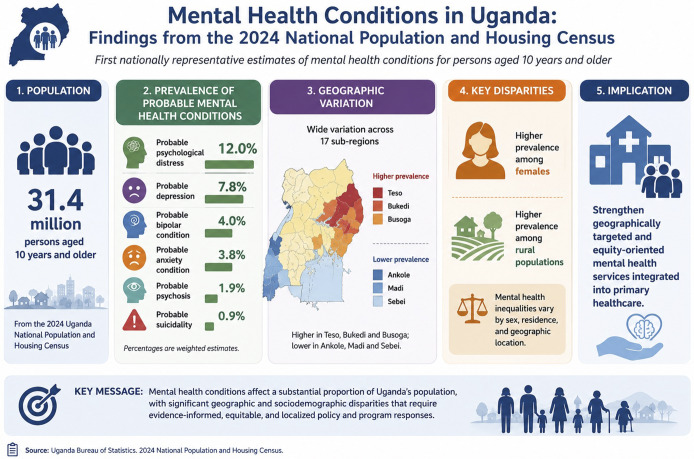

## Impact statement

Reliable national data on mental health are scarce in many low- and middle-income countries, making it difficult for governments to plan equitable services. Uganda’s 2024 national population census is an important step forward because it included simple questions on mental health symptoms across the population. Using these data, this study describes the distribution of probable general psychological distress, depression, anxiety, bipolar disorder, psychosis and suicidality among people aged 10 years and above. The findings show that mental health challenges affect a substantial proportion of the population and vary across regions and between rural and urban communities. This highlights that mental health is not only a clinical issue but also a population-level public health concern, requiring community-based and geographically targeted responses rather than uniform national approaches. Importantly, the study highlights the value of integrating mental health indicators into national data systems. Census-based measurement allows governments to identify higher-prevalence areas and plan services more equitably. At the same time, future censuses and large surveys could build on this progress by incorporating brief, internationally validated screening tools and additional indicators of risk and well-being to improve accuracy and policy relevance. Overall, this work shows that national censuses can play a major role in strengthening mental health surveillance and guiding evidence-based planning in resource-limited settings.

## Background

Globally, mental health disorders are among the leading causes of disability affecting over one billion people, with depression and anxiety being the most prevalent (Whiteford et al., 2013; World Health Organization., 2025). An estimated 80% of people with mental health conditions live in low- and middle-income countries (LMICs), where the burden is high (Ojagbemi and Gureje, 2022). In Africa, around 150 million people are affected amid limited mental health care services, with an increasing burden (World Health Organization, 2022). A similar increase in the burden of mental health disorders is observed across Sub-Saharan Africa, including East African countries (Charlson et al., 2014; Gouda et al., 2019; Kalungi et al., 2025) where a substantial proportion of these conditions are prevalent among young people (Jörns-Presentati et al., 2021; Ainamani et al., 2022, 2025b). Despite growing research, efforts to generate comprehensive statistical estimates of mental health conditions for different countries in the African region remain limited (Casella et al., 2025). Much of the existing evidence is derived from cross-sectional studies and documentary reviews (Opio et al., 2022; Kumar et al., 2024) resulting in fragmented population-level data that are inadequate for robust evidence-based planning and policy development.

In Uganda, mental health has historically received limited attention within national health priorities and financing frameworks (Ssebunnya et al., 2010; Kaggwa et al., 2022). Existing evidence is largely drawn from small community surveys, facility-based studies, or programme-specific evaluations that are geographically limited and not nationally representative (Opio et al., 2022).

Although these studies consistently indicate a high burden of mental health conditions, they do not provide a comprehensive picture of population-level prevalence (Kaggwa et al., 2022). The lack of nationally representative mental health data constrains effective planning, limits understanding of geographic and social inequalities, and hinders integration into primary healthcare at scale (Dwanyen et al., 2024). In response to this gap, Uganda recently conducted the 2024 National Population and Housing Census, marking a significant advancement in national mental health surveillance (Uganda Bureau of Statistics, 2024). The inclusion of mental health questions among persons aged 10 years and older represents an important step towards strengthening population-level mental health surveillance in Uganda. The Uganda Bureau of Statistics (UBOS) synthesised these responses into indicators of probable general psychological distress and five specific probable mental health conditions of depression, anxiety, bipolar disorder, psychosis and suicidality (Uganda Bureau of Statistics, 2024). This provides an unprecedented, nationally representative snapshot of mental health conditions across Uganda’s population, regions and urban–rural settings. This study aimed to describe the prevalence and distribution of probable mental health conditions among persons aged 10 years and older in Uganda, and to examine variations by sex, residence and sub-region using publicly available data from the 2024 National Population and Housing Census.

## Methods

### Study design and data source

This study was a descriptive secondary analysis of published data from the 2024 Uganda National Population and Housing Census, conducted by the Uganda Bureau of Statistics (Uganda Bureau of Statistics, 2024). The Uganda Bureau of Statistics (UBOS) is the national statistics authority established under the Uganda Bureau of Statistics Act 1998 and is mandated to coordinate the national statistics system and collect, analyze, and disseminate official statistics for Uganda. The national census is a core component of this mandate and represents the most comprehensive and authoritative source of population-level data in the country. The 2024 census was conducted nationwide using standardised instruments and procedures to ensure complete coverage and comparability across geographic and sociodemographic groups.

### Study population

The analytic population comprised 31,389,721 persons aged 10 years and older, drawn from the 2024 Uganda National Population and Housing Census. Uganda is a low-income country in East Africa with a predominantly young population and substantial geographic, socio-economic and cultural diversity (Uganda Bureau of Statistics, 2024; World Bank, 2024).

Traditionally, the country is divided into four broad regions, such as Central, Eastern, Northern and Western, which are further subdivided into 17 sub-regions used for planning, service delivery and statistical reporting by the Uganda Bureau of Statistics (Uganda Bureau of Statistics, 2024). Population distribution varies considerably across these regional blocks, with higher population concentration in the Central and Eastern regions, particularly within and around the Greater Kampala Metropolitan Area, and lower population density in parts of the Northern and north-eastern regions (Uganda Bureau of Statistics, 2024). Population distribution varies across these regions, with higher concentrations in the Central and Eastern regions, particularly around the Greater Kampala Metropolitan Area, and lower densities in parts of the Northern and north-eastern regions.

Uganda’s population is predominantly rural, with livelihoods in many areas dependent on subsistence agriculture and informal economic activities that are sensitive to climatic and economic shocks (Advocates Coalition for Development and Environment, 2019; United Nations Development Programme, 2022). Access to health and social services, including mental health services, varies across regions, reflecting differences in infrastructure development, urbanisation and historical patterns of public investment (Ssebunnya et al., 2010; Kaggwa et al., 2022). Mental health questions in the census were administered to all eligible individuals aged 10 years and above across all regions and sub-regions of the country, ensuring national coverage and enabling comparison of mental health indicators across geographic and socio-demographic contexts (Uganda Bureau of Statistics, 2024).

## Measurement of mental health indicators

Mental health status in the census was assessed using self-reported experiences of five symptoms: persistent sadness, persistent anxiety or excessive worry, hearing voices or seeing things that others do not, extreme mood swings and suicidal thoughts or plans. Responses to these items were recorded as part of the census questionnaire and are understood to reflect binary (yes/no) responses, consistent with standard census formats. Based on these items, the Uganda Bureau of Statistics, using publicly available census data, constructed six indicators of probable mental health conditions. Probable general psychological distress was defined as reporting at least one of the assessed symptoms. Specific probable conditions were defined as follows: probable depressive condition (persistent sadness), probable anxiety condition (persistent anxiety or excessive worry), probable bipolar condition (extreme mood swings), probable psychosis (hearing voices or seeing things that others do not) and probable suicidality (suicidal thoughts or plans). These indicators are derived from symptom-based responses and do not represent clinically validated diagnoses, but rather serve as screening-based proxies for population-level mental health conditions.

## Data analysis

Data were extracted from publicly available, aggregated tables published by the Uganda Bureau of Statistics as part of the 2024 Uganda National Population and Housing Census report. The analysis was descriptive and based on counts and percentages. Prevalence estimates were calculated as the proportion of individuals reporting each mental health symptom indicator among the total population aged 10 years and above. For subgroup analyses, percentages were computed within each category (sex, place of residence and sub-region), using the corresponding subgroup population as the denominator. Numerators represent the number of individuals reporting a given symptom, while denominators represent the relevant population group. Because the analysis relied on aggregated census data, individual-level analysis and inferential statistical testing were not conducted. The findings should therefore be interpreted as descriptive population-level estimates rather than measures of association or causation. Missing data for mental health indicators were not observed in the published census tables.

## Results

### Descriptive characteristics

The analytic population comprised 31,389,721 persons aged 10 years and above. Females constituted a larger proportion of the population than males (17,134,039 [54.6%] vs. 14,255,682 [45.4%]). The majority of the population resided in rural areas (19,537,803 [62.2%]), compared with urban areas (11,851,918 [37.8%]). Distribution across sub-regions varied, with the largest populations in Buganda (7,527,110 [24.0%]) and Busoga (2,972,581 [9.5%]), and smaller proportions in sub-regions such as Sebei (262,072 [0.8%]) and Madi (379,606 [1.2%]). Detailed population characteristics are presented in Table 1 and Supplementary Figure 1.

**Table 1.** Descriptive characteristics of persons aged 10 years and above, Uganda (*N* = 31,389,721) Table 1. long description.

Population characteristic	n	%
Sex		
Male	14,255,682	45.4
Female	17,134,039	54.6
Residence		
Urban	11,851,918	37.8
Rural	19,537,803	62.2
Sub-region		
Kampala Capital City	1,190,702	3.8
Buganda	7,527,110	24.0
Busoga	2,972,581	9.5
Bukedi	1,623,382	5.2
Bugisu	1,306,879	4.2
Sebei	262,072	0.8
Teso	1,657,175	5.3
Karamoja	919,324	2.9
Lango	1,811,140	5.8
Acholi	1,424,752	4.5
West Nile	2,247,786	7.2
Madi	379,606	1.2
Bunyoro	1,892,351	6.0
Tooro	1,467,296	4.7
Rwenzori	831,527	2.6
Ankole	2,644,596	8.4
Kigezi	1,231,442	3.9
Total	31,389,721	100.0

## National prevalence estimates of all probable mental health conditions

Differences in probable mental health conditions were observed by sex in gender. Females reported a higher prevalence of probable general psychological distress compared with males (2,127,535 [12.4%] vs. 1,646,380 [11.5%]). Females also had a higher prevalence of probable depressive condition (1,393,581 [8.1%] vs. 1,050,691 [7.4%]) and probable anxiety condition (685,976 [4.0%] vs. 515,206 [3.6%]). The prevalence of probable bipolar condition was also higher among females compared with males (~4.2% vs. 3.8%). Prevalence of probable psychosis (~2.0% vs. 1.8%) and probable suicidality (150,921 [0.9%] vs. 121,350 [0.9%]) was similar between females and males. Detailed estimates are presented in Table 2.

**Table 2.** National prevalence of all probable mental health conditions among persons aged 10 years and above, Uganda (*N* = 31,389,721) Table 2. long description.

Mental health conditions	*n*	*%*
Probable general psychological distress	3,773,915	12.0
Probable depressive condition	2,444,272	7.8
Probable anxiety condition	1,201,182	3.8
Probable bipolar condition	1,262,088	4.0
Probable psychosis	584,969	1.9
Probable suicidality	272,271	0.9

*Note:* Percentages are calculated using the total population aged 10 years and above as the denominator. Mental health indicators represent probable conditions based on self-reported symptoms. Table 2 for further illustration.

## Probable mental health conditions by gender

Differences in probable mental health conditions were observed by sex. Females reported a higher prevalence of probable general psychological distress than males (2,127,535 [12.4%] vs. 1,646,380 [11.5%]). Females also had a higher prevalence of probable depressive condition (1,393,581 [8.1%] vs. 1,050,691 [7.4%]) and probable anxiety condition (685,976 [4.0%] vs. 515,206 [3.6%]). Prevalence of probable suicidality was similar among females and males (150,921 [0.9%] vs. 121,350 [0.9%]). Full sex-disaggregated estimates are presented in Table 3.

**Table 3.** Probable mental health conditions by sex (*n*, %) Table 3. long description.

Condition	Male*n* (%)	Female*n* (%)
Psychological distress	1,646,380 (11.5)	2,127,535 (12.4)
Depression	1,050,691 (7.4)	1,393,581 (8.1)
Anxiety	515,206 (3.6)	685,976 (4.0)
Bipolar	559,470 (3.9)	702,618 (4.1)
Psychosis	259,102 (1.8)	325,867 (1.9)
Suicidality	121,350 (0.9)	150,921 (0.9)

*Note:* Percentages (%) are calculated within sex (male or female). Mental health indicators represent probable conditions based on self-reported symptoms.

## Probable mental health conditions by residence

By place of residence, differences in probable mental health conditions were observed. Rural residents reported a higher prevalence of probable general psychological distress compared with urban residents (2,479,535 [12.7%] vs. 1,294,380 [10.9%]). Rural populations also had higher prevalence of probable depressive condition (1,576,322 [8.1%] vs. 867,950 [7.3%]), probable anxiety condition (790,763 [4.0%] vs. 410,419 [3.5%]) and probable bipolar condition (855,756 [4.4%] vs. 406,332 [3.4%]). Prevalence of probable psychosis (~2.0% vs. 1.7%) and probable suicidality (~1.0% vs. 0.8%) was also higher in rural compared with urban areas. Detailed estimates are presented in Table 4.

**Table 4.** Probable mental health conditions by residence (*n*, %) Table 4. long description.

Condition	Urban*n* (%)	Rural*n* (%)
Psychological distress	1,294,380 (10.9)	2,479,535 (12.7)
Depression	867,950 (7.3)	1,576,322 (8.1)
Anxiety	410,419 (3.5)	790,763 (4.0)
Bipolar	406,332 (3.4)	855,756 (4.4)
Psychosis	187,487 (1.6)	397,482 (2.0)
Suicidality	87,704 (0.7)	184,567 (0.9)

*Note:* Percentages (%) are calculated out of the total population aged 10 years and above.

## Sub-regional variation in probable mental health conditions

Substantial variation in probable general psychological distress was observed across Uganda’s sub-regions. Prevalence ranged from 21,487 (8.1%) in Sebei and 30,325 (8.2%) in Madi to 271,375 (16.4%) in Teso. Other sub-regions with relatively higher prevalence included Bukedi (254,630 [15.7%]), Bugisu (197,307 [15.1%]) and Busoga (424,453 [14.3%]), while lower prevalence was observed in Ankole (75,000 [8.8%]), Kampala (91,268 [9.0%]) and Buganda (711,342 [9.5%]). Patterns for specific probable mental health conditions showed similar geographic variation across sub-regions. Details are presented in Table 5.

**Table 5.** Sub-regional distribution of probable mental health conditions among persons aged 10 years and above, Uganda Table 5. long description.

Sub-region	Psychological distress (%)	Depression*n* (%)	Anxiety*n* (%)	Bipolar*n* (%)	Psychosis*n* (%)	Suicidality*n* (%)
Teso	271,375 (16.4)	182,911 (11.1)	96,741 (5.9)	108,902 (6.6)	51,237 (3.1)	23,845 (1.4)
Bukedi	254,630 (15.7)	171,204 (10.6)	89,327 (5.5)	102,114 (6.3)	48,956 (3.0)	22,641 (1.4)
Bugisu	197,307 (15.1)	131,882 (10.1)	71,946 (5.5)	81,463 (6.2)	39,211 (3.0)	17,993 (1.4)
Busoga	424,453 (14.3)	287,694 (9.7)	151,928 (5.1)	170,214 (5.7)	82,416 (2.8)	36,478 (1.2)
Karamoja	156,142 (13.6)	101,893 (8.9)	57,206 (5.0)	63,114 (5.5)	32,984 (2.9)	15,246 (1.3)
Acholi	170,548 (12.3)	115,604 (8.4)	63,291 (4.6)	69,483 (5.0)	35,812 (2.6)	16,118 (1.2)
Lango	182,991 (12.0)	123,876 (8.1)	65,914 (4.3)	73,184 (4.8)	36,701 (2.4)	16,842 (1.1)
West Nile	189,204 (11.8)	127,511 (7.9)	67,482 (4.2)	75,309 (4.7)	37,994 (2.4)	17,216 (1.1)
Elgon	80,476 (11.3)	54,211 (7.6)	28,993 (4.1)	32,845 (4.6)	16,194 (2.3)	7,241 (1.0)
Bunyoro	130,762 (10.9)	87,194 (7.2)	45,682 (3.8)	50,963 (4.2)	25,381 (2.1)	11,674 (1.0)
Tooro	145,923 (10.7)	97,845 (7.1)	50,764 (3.7)	55,314 (4.0)	27,614 (2.0)	12,483 (0.9)
Kigezi	154,682 (9.8)	103,116 (6.6)	53,987 (3.4)	58,611 (3.7)	29,407 (1.9)	13,215 (0.8)
Buganda	711,342 (9.5)	460,871 (6.1)	235,991 (3.1)	259,814 (3.5)	124,932 (1.7)	53,724 (0.7)
Kampala	91,268 (9.0)	58,417 (5.8)	29,984 (3.0)	33,117 (3.3)	15,834 (1.6)	6,998 (0.7)
Ankole	75,000 (8.8)	49,000 (5.8)	25,000 (2.9)	27,000 (3.2)	12,000 (1.4)	5,000 (0.6)
Sebei	21,487 (8.1)	14,268 (5.4)	7,326 (2.8)	8,014 (3.0)	3,917 (1.5)	1,724 (0.7)
Madi	30,325 (8.2)	20,071 (5.4)	10,284 (2.8)	11,613 (3.2)	5,782 (1.6)	2,463 (0.7)

*Note:* Percentages (%) are calculated within each sub-region. Sub-regions are defined according to the Uganda Bureau of Statistics administrative classification. Mental health indicators represent probable conditions based on self-reported symptoms.

## Discussion

This study provides the first nationally representative description of probable mental health conditions among persons aged 10 years and older in Uganda using data from the 2024 National Population and Housing Census. At the national level, approximately one in eight persons (12%) experienced probable general psychological distress corresponding to nearly four million people. Among specific probable mental health conditions, probable depressive disorder was the most prevalent (7.8%), followed by probable bipolar condition (4.4%) and probable anxiety condition (3.8%). Probable psychosis affected about 1.9%, while probable suicidality was reported by 272,271 individuals (0.9%).

The observed prevalence of probable general psychological distress (3,773,915 (12.0%) and specific conditions such as depression, anxiety and bipolar disorder is broadly consistent with evidence from other low- and middle-income countries, where common mental health conditions are widely reported in the general population (Kaggwa et al., 2022; Opio et al., 2022; Kalungi et al., 2025).

This level of population exposure indicates that mental health problems are not confined to small or clinically visible groups but represent a broad public health concern with potential implications for daily functioning, social participation, quality of life and economic productivity (Michon et al., 2008; Singh et al., 2015; Immurana et al., 2024). Similar population-level burdens of psychological distress and common mental disorders have been documented across sub-Saharan Africa and East Africa, where community-based studies and national surveys consistently report high prevalence of depressive and anxiety-related symptoms in the general population (Greene et al., 2021; Jörns-Presentati et al., 2021; Gbadamosi et al., 2022). In Uganda, previous national estimates have suggested that up to 14 million people may be living with a mental health condition, based largely on extrapolations from smaller studies and service utilisation data rather than nationally representative population measurement (Kaggwa et al., 2022; Opio et al., 2022).

While these earlier estimates are not directly comparable to the census-based findings presented here due to differences in age coverage, definitions and measurement approaches – they nevertheless suggest that mental health challenges are present at a notable level in the population. The present findings, based on nationally representative census data among persons aged 10 years and above and derived from standardised symptom indicators, extend this evidence by providing population-level estimates of probable mental health conditions in Uganda.

Beyond general psychological distress, the prevalence of specific probable mental health conditions observed in the census is broadly consistent with patterns reported in prior community-based and facility-based studies in Uganda (Kaggwa et al., 2022; Opio et al., 2022; Mugisha et al., 2025; Ziegel et al., 2025). Studies conducted in different regions of the country have documented high levels of psychotic features, suicidality and symptoms of bipolar mental health conditions among adolescents and adults, often exceeding estimates from high-income settings, although such studies have typically been geographically limited and methodologically heterogeneous(Rukundo et al., 2016; Mwesiga et al., 2020; Ainamani et al., 2025a). The relatively lower prevalence of probable psychosis and suicidality observed in the census mirrors findings from community surveys in Uganda and the wider East African region, where severe mental health conditions are less common at the population level but associated with substantial morbidity, mortality and social exclusion (Bentall et al., 2007; Lundberg et al., 2009; Mwesiga et al., 2020; Bahati, Ainamani, et al., 2022; Bonnell et al., 2022). Taken together, these findings highlight the value of including mental health modules in national censuses and support the inclusion of expanded and standardised mental health measures in future population-based assessments to improve comparability and inform targeted interventions.

Our synthesis of census data indicates consistent gender differences across all assessed probable mental health conditions, with higher prevalence reported among females than males. This pattern was observed for probable general psychological distress, as well as for all specific conditions assessed, including probable depression, anxiety, bipolar disorder, psychosis and suicidality, although the magnitude of differences varied by condition.

The higher prevalence of psychological distress and common mental health mentions among females is consistent with findings from population-based studies across Uganda, East Africa and sub-Saharan Africa, where women tend to report higher levels of mental health-related symptoms than men (Mugisha et al., 2025; Ziegel et al., 2025). For example, evidence from Uganda indicates notable gender differences in the presentation of psychotic conditions. A 1-year prevalence study among first-treatment-contact patients at the National Psychiatric Referral and Teaching Hospital found that women exhibited more pronounced psychotic features compared to men (Bentall et al., 2007; Mwesiga et al., 2020; Ceasar Kimera et al., 2024). Similarly, prior evidence shows that women experience a disproportionate burden of common mental conditions, particularly depression and anxiety, which are closely linked to social and structural determinants of health (Lund et al., 2018; Ainamani et al., 2022; Cheng et al., 2025).

In the Ugandan context, several factors may contribute to these observed gender differences. Women and girls are more likely to experience caregiving burdens, economic insecurity, gender-based violence, sexual violence and constrained access to education and employment opportunities, all of which are established risk factors for psychological distress (Altemus et al., 2014; Kaggwa et al., 2021; Ainamani et al., 2022). Biological and social vulnerabilities associated with gender may accumulate across the life course, potentially increasing exposure to chronic stress and mental health challenges (Altemus et al., 2014). At the same time, gender norms may influence help-seeking behaviour and symptom reporting, with men potentially underreport psychological distress due to stigma and expectations around masculinity, a pattern observed in other African settings and across the globe (Kobusingye et al., 2025; Burns et al., 2026). Importantly, although gender differences were modest in absolute terms, their consistency across all assessed mental health outcomes highlights the need for gender-responsive mental health approaches (Kobusingye et al., 2025). Integrating mental health services into platforms that routinely engage women, such as primary healthcare, maternal and reproductive health services and community-based programmes, may help address women’s disproportionate exposure to psychosocial stressors. At the same time, strategies to improve mental health awareness and help-seeking among men are also needed to ensure equitable access to care.

Our synthesis of census data indicates consistently higher prevalence of probable mental health conditions among rural residents compared with urban residents across all assessed outcomes, highlighting place of residence as a key dimension of mental health inequality in Uganda. These findings are consistent with the community studies in Uganda that have previously found higher levels of psychological distress in rural settings than urban communities (Opio et al., 2022; Seruwagi et al., 2022). Higher levels of psychological distress in rural areas may reflect the cumulative effects of structural and environmental stressors that disproportionately affect rural populations, including poverty, limited access to services and environmental vulnerability (Ndyanabangi et al., 2004; Kigozi et al., 2010).

In Uganda, rural communities are more likely to experience chronic poverty, food insecurity, livelihood instability and exposure to climate-related shocks such as droughts and flooding (Lawrance et al., 2022; Mokhwelepa and Sumbane, 2025). These stressors have been consistently linked to elevated risk of common mental health disorders in low- and middle-income countries, particularly in predominantly agrarian settings (Atwoli et al., 2022; Mahmood et al., 2025).

Limited access to health and social services in rural areas may further exacerbate mental health conditions (Kigozi et al., 2010). For example, mental health services in Uganda remain heavily concentrated in urban centres, and rural populations often face long travel distances, shortages of trained personnel and limited availability of psychosocial support (Kigozi et al., 2010; Dwanyen et al., 2024).

Conversely, although urban residents may experience distinct stressors, they may benefit from greater access to healthcare facilities, educational opportunities, employment and social services, which can mitigate some mental health risks (Kigozi et al., 2010; Katayama et al., 2023). The observed urban–rural disparities underscore the importance of strengthening mental health integration within primary healthcare in rural areas, including task-sharing approaches, community-based psychosocial support and improved referral systems.

Our findings indicate pronounced sub-regional variation in probable general psychological distress across Uganda, suggesting that the mental health conditions are unevenly distributed geographically. Higher prevalence was observed in sub-regions such as Teso, Bukedi, Bugisu and Busoga, whereas comparatively lower prevalence was recorded in Ankole and Buganda. These patterns highlight the importance of regional context in shaping population mental health outcomes.

Several of the high-burden sub-regions identified are located in eastern and parts of northern Uganda, areas that have historically experienced economic marginalisation, livelihood insecurity and repeated environmental shocks (Kabunga et al., 2022; Batte et al., 2024; Kazibwe, 2025). These areas are increasingly exposed to climate-related shocks and mobility pressures, which may compound existing social and economic stressors and may contribute to elevated psychological distress (International Organization for Migration, 2023). Evidence from sub-Saharan Africa consistently demonstrates strong associations between socio-economic adversity and common mental health conditions, particularly in agrarian and climate-vulnerable settings (Atwoli et al., 2022; Mahmood et al., 2025). By contrast, sub-regions such as Buganda and Ankole, which exhibited lower prevalence of probable psychological distress, generally benefit from relatively better infrastructure, economic opportunities and access to health and social services. The lower prevalence observed in these regions may be associated with greater availability of education, employment and healthcare services, which could help reduce chronic stressors and improve access to support. However, these geographic differences should be interpreted with caution, as they may be influenced by reporting practices, cultural variation in the expression of distress or unmeasured contextual factors. In addition, the observed patterns may be subject to ecological fallacy, whereby associations observed at the group level may not reflect individual-level relationships.

Taken together, these regional imbalances underscore the importance of geographically targeted mental health strategies rather than uniform national approaches. Population-level evidence from the census provides a critical foundation for identifying high-burden sub-regions and prioritising resource allocation. Strengthening community-based psychosocial support, integrating mental health into primary healthcare and expanding outreach services in high-prevalence regions may be particularly important for reducing regional mental health inequalities. These implications align with global mental health recommendations that emphasise context-specific, equity-oriented approaches to mental health system strengthening in low- and middle-income countries.

### Strengths and limitations

This study has several important strengths. Most notably, it draws on nationally representative census data, providing the first population-wide description of probable mental health conditions in Uganda among persons aged 10 years and older. The inclusion of mental health indicators in the 2024 National Population and Housing Census represents a major advance for mental health surveillance, enabling assessment of prevalence, distribution and inequalities across sex, residence and sub-regions at an unprecedented scale.

However, several limitations should be acknowledged. First, mental health outcomes were based on self-reported symptom indicators rather than clinical diagnoses. The indicators, therefore, represent probable mental health conditions and should be interpreted as markers of psychological distress at the population level rather than definitive clinical conditions. Second, the analysis relied exclusively on aggregated, published census tabulations, which limited the ability to conduct inferential statistical analyses, assess statistical significance or adjust for potential confounders. Third, the census mental health module included a limited number of symptom-based questions and did not incorporate standardised diagnostic screening instruments, which may affect comparability with other epidemiological studies. Fourth, the census did not specify a defined recall period for symptom reporting, which may influence the interpretation of prevalence estimates. Fifth, responses may include both self-reported and proxy-reported information, which could introduce reporting bias. Sixth, mental health data were collected only for persons aged 10 years and older, precluding assessment among younger children. In addition, observed geographic differences may be influenced by reporting patterns, cultural interpretation of symptoms, or contextual factors and should therefore be interpreted with caution (ecological fallacy).

Future population-based data collection efforts could consider including younger children below 10 years of age, alongside the incorporation of brief, standardised and age-appropriate screening tools that capture symptom severity, to provide a more comprehensive understanding of mental health across the life course.

## Conclusion

Using nationally representative data from the 2024 Uganda National Population and Housing Census, this study provides evidence of a substantial and uneven distribution of probable mental health conditions among persons aged 10 years and older in Uganda. Approximately one in eight individuals reported probable general psychological distress, with consistent variations observed by sex, residence and sub-region. These findings underscore that mental health challenges are widespread and may be associated with structural and geographic inequalities.

The observed gender, urban–rural and sub-regional disparities highlight the need for equity-oriented, geographically targeted mental health strategies, particularly in higher-prevalence and underserved areas. Strengthening the integration of mental health services into primary healthcare and community systems may be important for addressing mental health inequalities and improving population mental health outcomes in Uganda. The 2024 Census provides a critical evidence base to guide these efforts and sets an important precedent for future population-based mental health surveillance.

Future censuses and national surveys may benefit from including younger children below 10 years of age and incorporating brief, validated and age-appropriate screening tools that assess symptom severity, to enhance the accuracy, comparability and policy relevance of population-level mental health data.

## Supporting information

10.1017/gmh.2026.10237.sm001 Ainamani et al. supplementary material Ainamani et al. supplementary material

## Data Availability

The data used in this study are publicly available and can be accessed through the Uganda Bureau of Statistics 2024 National Population and Housing Census reports.

## Supplementary material

The supplementary material for this article can be found at http://doi.org/10.1017/gmh.2026.10237.

## Table 1. Long description

The table is organized in three main sections. The first section, Sex, lists Male with 14,255,682 individuals at 45.4 percent and Female with 17,134,039 at 54.6 percent. The next section, Residence, shows Urban with 11,851,918 at 37.8 percent and Rural with 19,537,803 at 62.2 percent. The final section, Sub-region, lists Kampala Capital City with 1,190,702 at 3.8 percent, Buganda with 7,527,110 at 24.0 percent, Busoga with 2,972,581 at 9.5 percent, Bukedi with 1,623,382 at 5.2 percent, Bugisu with 1,306,879 at 4.2 percent, Sebei with 262,072 at 0.8 percent, Teso with 1,657,175 at 5.3 percent, Karamoja with 919,324 at 2.9 percent, Lango with 1,811,140 at 5.8 percent, Acholi with 1,424,752 at 4.5 percent, West Nile with 2,247,786 at 7.2 percent, Madi with 379,606 at 1.2 percent, Bunyoro with 1,892,351 at 6.0 percent, Tooro with 1,467,296 at 4.7 percent, Rwenzori with 831,527 at 2.6 percent, Ankole with 2,644,596 at 8.4 percent, and Kigezi with 1,231,442 at 3.9 percent. The total population is 31,389,721, representing 100.0 percent.

Navigate back to Table 1..

## Table 2. Long description

The table has three columns: Mental health conditions, n, and percent. From top to bottom, the rows list: Probable general psychological distress with 3,773,915 cases at 12.0 percent; probable depressive condition with 2,444,272 cases at 7.8 percent; probable anxiety condition with 1,201,182 cases at 3.8 percent; probable bipolar condition with 1,262,088 cases at 4.0 percent; probable psychosis with 584,969 cases at 1.9 percent; and probable suicidality with 272,271 cases at 0.9 percent. Percentages are calculated using the total population aged 10 years and above as the denominator. All indicators represent probable conditions based on self-reported symptoms.

Navigate back to Table 2..

## Table 3. Long description

Starting from the top row, the table lists six mental health conditions. For psychological distress, males have 1,646,380 cases (11.5 percent), females 2,127,535 (12.4 percent). Depression shows 1,050,691 males (7.4 percent), 1,393,581 females (8.1 percent). Anxiety is 515,206 males (3.6 percent), 685,976 females (4.0 percent). Bipolar disorder is 559,470 males (3.9 percent), 702,618 females (4.1 percent). Psychosis is 259,102 males (1.8 percent), 325,867 females (1.9 percent). Suicidality is 121,350 males (0.9 percent), 150,921 females (0.9 percent). Percentages are calculated within each sex. Across all conditions, female counts and percentages are higher than male values.

Navigate back to Table 3..

## Table 4. Long description

The table has three columns: Condition, Urban n (percent), and Rural n (percent). From top to bottom, the rows are: Psychological distress, Urban 1,294,380 (10.9), Rural 2,479,535 (12.7); Depression, Urban 867,950 (7.3), Rural 1,576,322 (8.1); Anxiety, Urban 410,419 (3.5), Rural 790,763 (4.0); Bipolar, Urban 406,332 (3.4), Rural 855,756 (4.4); Psychosis, Urban 187,487 (1.6), Rural 397,482 (2.0); Suicidality, Urban 87,704 (0.7), Rural 184,567 (0.9). For each condition, both the count and percentage are higher in rural than urban populations. Percentages are calculated from the total population aged 10 years and above.

Navigate back to Table 4..

## Table 5. Long description

From the top row, each sub-region is listed in the first column, followed by six columns for mental health indicators. For psychological distress, Teso has 271,375 cases (16.4 percent), Bukedi 254,630 (15.7 percent), Bugisu 197,307 (15.1 percent), Busoga 424,453 (14.3 percent), Karamoja 156,142 (13.6 percent), Acholi 170,548 (12.3 percent), Lango 182,991 (12.0 percent), West Nile 189,204 (11.8 percent), Elgon 80,476 (11.3 percent), Bunyoro 130,762 (10.9 percent), Tooro 145,923 (10.7 percent), Kigezi 154,682 (9.8 percent), Buganda 711,342 (9.5 percent), Kampala 91,268 (9.0 percent), Ankole 75,000 (8.8 percent), Sebei 21,487 (8.1 percent), Madi 30,325 (8.2 percent). Depression rates are highest in Teso 182,911 (11.1 percent) and Bukedi 171,204 (10.6 percent), lowest in Sebei 14,268 (5.4 percent) and Madi 20,071 (5.4 percent). Anxiety, bipolar, psychosis, and suicidality follow similar patterns, with Teso, Bukedi, and Bugisu consistently higher, and Sebei, Madi, and Ankole lower. For suicidality, Teso has 23,845 (1.4 percent), Bukedi 22,641 (1.4 percent), Bugisu 17,993 (1.4 percent), while Sebei has 1,724 (0.7 percent), Madi 2,463 (0.7 percent), and Ankole 5,000 (0.6 percent). Percentages are calculated within each sub-region. Sub-regions are defined by the Uganda Bureau of Statistics. All indicators are based on self-reported symptoms.

Navigate back to Table 5..
