# Supplementary material for: Prevalence and regional disparities in probable mental health conditions among persons aged 10 years and older in Uganda: Evidence from the 2024 National Population and Housing Census
Source: Glob Ment Health (Camb). 2026 May 28;13:e122. doi: 10.1017/gmh.2026.10237 (PMC13279965; doi:10.1017/gmh.2026.10237)

***Supplimentary Figure1* : Percentage of Persons aged 10 and above with Probable General Psychological Distress by sub Region**


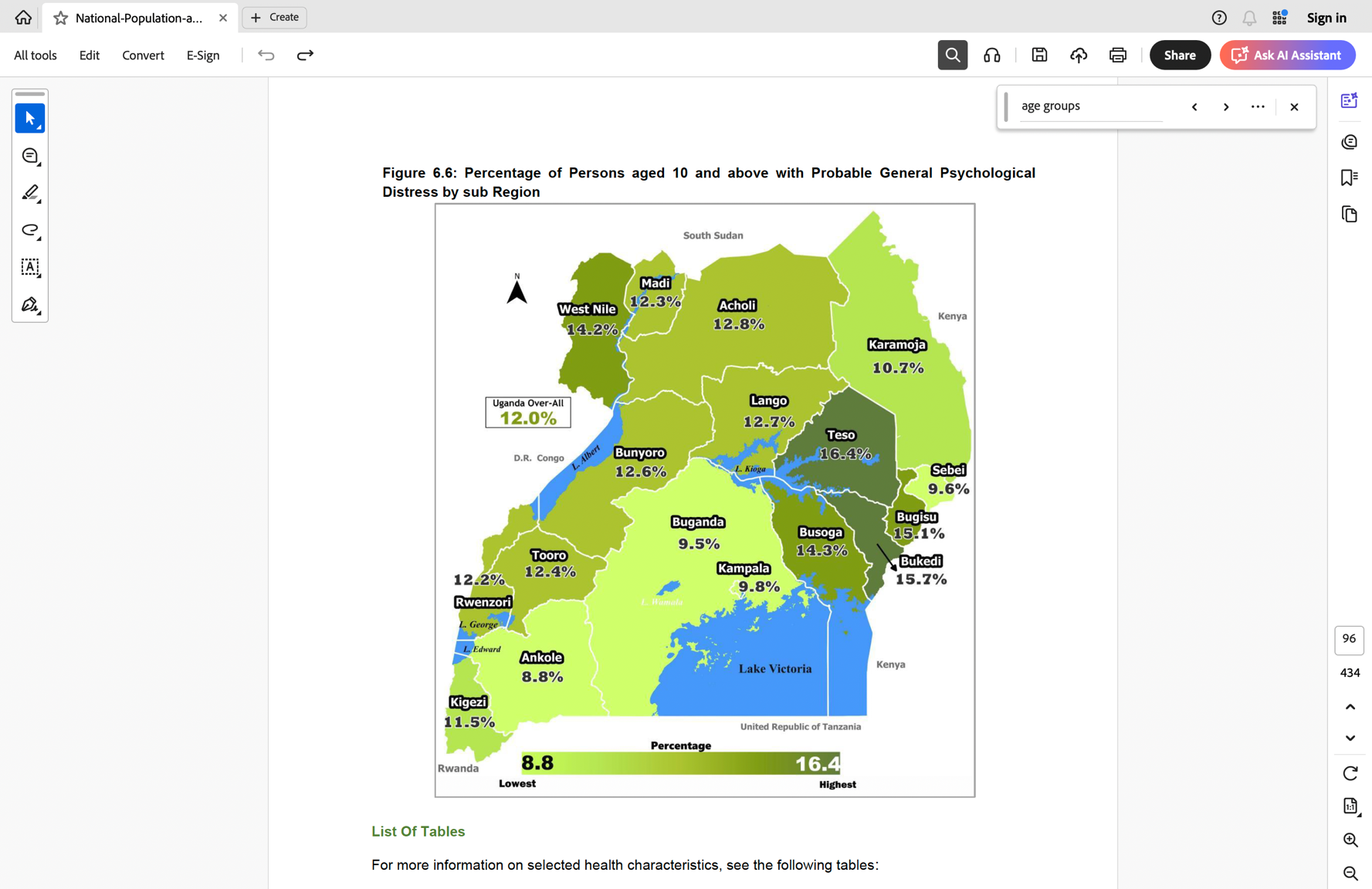

Supplement: Ainamani et al. supplementary material [file S2054425126102374sup001.docx]
