# Supplementary material for: Prevalence and regional disparities in probable mental health conditions among persons aged 10 years and older in Uganda: Evidence from the 2024 National Population and Housing Census
Source: Glob Ment Health (Camb). 2026 May 28;13:e122. doi: 10.1017/gmh.2026.10237 (PMC13279965; doi:10.1017/gmh.2026.10237)
Supplement: Peer review history

# Reviewer report: Review: Prevalence and regional disparities in probable mental health conditions among persons aged 10 years and older in Uganda: Evidence from the 2024 National Population and Housing Census — R0/PR2

**DOI:** 10.1017/gmh.2026.10237.pr2

This study uses the 2024 Uganda National Population and Housing Census to analyze the prevalence and sociodemographic disparities of probable mental health disorders among people aged 10 years and older. The research topic is highly relevant to global mental health in low- and middle-income countries, the data are nationally representative and authoritative, the study design is appropriate, and the conclusions are clear. The manuscript is generally well-written. Addressing the comments below will strengthen methodological transparency, interpretability, consistency, and policy utility.

Suggestions for Revision:

1.The manuscript uses inconsistent terminology, alternating between “probable mental health disorders” and “probable conditions,” which may confuse readers about the clinical status of the identified cases. To ensure conceptual precision, consistency with WHO and mhGAP terminology, and clear differentiation between screening-based indicators and formal clinical diagnoses, I recommend using the term “probable mental health conditions” uniformly throughout the manuscript.

2.The manuscript states that mental health outcomes were “based on self‑reported symptoms synthesised by the Uganda Bureau of Statistics into six indicators.” However, specific instruments, item wording, coding, scoring algorithms, and thresholds for defining each “probable” condition are not provided. Without this detail, readers cannot evaluate validity, comparability with standardized tools (e.g., PHQ‑9, GAD‑7), or misclassification bias. Please add a dedicated subsection under Methods (e.g., “Measurement of Mental Health Indicators”) that clearly reports the exact census questions used for each symptom, response options (binary yes/no, Likert scale, etc.), how UBOS aggregated responses into the six probable mental health indicators, thresholds or criteria used to define caseness for each indicator with justification, and a reference to official UBOS technical documentation if available.

3.The study population includes individuals aged 10 years and above, but adolescents aged 10–18 years have unique developmental, cognitive, and cultural characteristics. The manuscript does not justify whether the same symptom questions are valid, developmentally appropriate, or interpretable for adolescents compared with adults. Please explicitly address whether the measurement items were validated or piloted for adolescents; if not, clearly discuss this limitation in the Methods or Limitations section.

4.The term “burden” is used repeatedly throughout the manuscript but is not appropriately justified. Disease burden represents a comprehensive summary measure of population health loss, including mortality, disability, quality of life, and socioeconomic impact, often quantified using metrics such as DALYs. This study only reports prevalence rates derived from self‑reported symptoms, with no data on severity, functional impairment, mortality, or economic consequences. Please replace or qualify “burden” in the title, abstract, and main text with more accurate and precise terms such as prevalence, caseload, frequency, population‑level symptoms, or reported distress.

5.The description of data analysis procedures is overly brief and lacks key transparency details. Please explicitly explain how prevalence rates were calculated, including clear definitions of numerators and denominators and whether percentages are computed for the total population or within specific subgroups. Please also report the proportion of missing data for mental health indicators and how missing values were handled during analysis.

6.The study covers individuals aged 10 years and older, encompassing adolescents, young adults, middle‑aged adults, and older adults—groups with distinctly different mental health risks and symptom patterns. Age is a well‑established critical determinant of mental health prevalence and expression, yet the manuscript provides no age‑stratified prevalence estimates and does not discuss age‑related disparities. Please clarify whether age‑stratified analyses were conducted. If such results are not included, explain the rationale and acknowledge this gap as an important limitation.

7.While the observed sub‑regional variation in general psychological distress is striking and policy‑relevant, the manuscript currently attributes this variation directly to regional differences in underlying prevalence without acknowledging the risk of ecological fallacy. Geographic differences may reflect heterogeneous case detection, reporting biases, cultural variation in expressing distress, or unmeasured contextual confounders rather than true differences in population prevalence. In the Discussion section, please explicitly acknowledge this limitation.

Thank you for the opportunity to review this important contribution to global mental health.

# Reviewer report: Review: Prevalence and regional disparities in probable mental health conditions among persons aged 10 years and older in Uganda: Evidence from the 2024 National Population and Housing Census — R0/PR3

**DOI:** 10.1017/gmh.2026.10237.pr3

Comments

Abstract

Major Comments

1. Could you clarify more explicitly that the outcomes are based on census-derived symptom indicators rather than clinically diagnosed mental disorders?

2. The phrase “probable mental health disorders” may be too strong given the measurement approach. Could this be reworded to better reflect symptom-based estimates?

3. The conclusion may sound more definitive than the data allow. Could you soften it slightly to reflect the descriptive nature of the study?

Minor Comments

4. The methods could briefly indicate how the mental health variables were constructed to improve clarity for readers.

Introduction

Major Comments

1. Some statements appear quite broad. Please make them more precise and clearly tied to the Ugandan context

2. It would help to clearly distinguish between mental health symptoms, screening indicators, and diagnosed disorders early in the introduction.

Minor Comments

3. Some claims would benefit from more recent or multiple references. Could you review and update the

4. citations where needed?

Methods

Major Comments

5. Please explain more clearly how individual symptom questions were used to define categories such as probable depression, anxiety, bipolar disorder, and psychosis

6. How were responses obtained? were they self-reported, proxy-reported, or both?

7. Clarify on whether there was a defined recall period for the symptom questions?

8. The definition of general psychological distress as the presence of at least one symptom seems broad. Could you explain the rationale and implications of this definition?

Minor Comments

9. Consider briefly noting any limitations related to translation or interpretation of questions across languages.

Results

Major Comments

10. Please ensure that all reported percentages are fully consistent between the text, tables, and figures.

11. Some descriptions of regional variation feel selective. Could you present the pattern more systematically across all regions?

12. Since the study includes individuals aged 10 years and above, could you include age-disaggregated findings or explain why they are not available?

Minor Comments

13. Some wording in this section sounds slightly interpretive. It may help to keep the language strictly descriptive.

Discussion

Major Comments

14. Some interpretations go beyond what the data can support. Consider revising the language to be more cautious throughout

15. Where differences by sex, region, or residence are discussed, could you present these as possible explanations rather than conclusions?

16. The explanation of regional differences is interesting but sometimes speculative. Could you focus more on evidence-based interpretations?

17. The suggestion linking lower burden to the presence of universities or referral hospitals may need to be reconsidered or better justified.

Minor Comments

18. When using terms such as “higher burden” or “longer,” could you define or contextualize them more clearly?

19. You may consider adding a short paragraph explaining the value of census data for mental health surveillance despite measurement limitations.

Limitations

Major Comments

20. The limitations section could be expanded. Could you discuss more explicitly the challenges of using census-based mental health indicators?

21. Consider addressing potential issues such as proxy reporting, stigma, and underreporting of symptoms?

Minor Comments

22. It would be helpful to clearly state that these findings should not be interpreted as clinical prevalence estimates.

Conclusion

Major Comments

23. The conclusions could be more closely aligned with the data presented. Avoid overly general statements

24. The recommendations are somewhat broad. It would be helpful to make them more specific and directly linked to your findings

Minor Comments

25. It would strengthen the paper to include clearer future research directions, such as qualitative or longitudinal studies.

Language and Referencing

26. There are several typographical and grammatical errors that should be corrected before publication.

27. Please check the reference list for duplicate entries and ensure consistent formatting.

28. It would also help to use terminology consistently throughout the manuscript when referring to symptoms versus disorders.

# Editor report: Recommendation: Prevalence and regional disparities in probable mental health conditions among persons aged 10 years and older in Uganda: Evidence from the 2024 National Population and Housing Census — R0/PR4

**DOI:** 10.1017/gmh.2026.10237.pr4

**Affiliations:** Psychology, UNISA, South Africa

Dear Dr. Ainamani,

Your manuscript, “Prevalence and regional disparities in probable mental health disorders among persons aged 10 years and older in Uganda: Evidence from the 2024 National Population and Housing Census”, has now been assessed. You will see that, while the reviewers find your work of interest, they have raised points that need to be addressed by a Major revision. 

We invite you to revise your paper, carefully addressing the comments from the reviewers and the editor. Please ensure the results are accurately reported, any overstated conclusions are rewritten, and the limitations of the work are fully explained. When your revision is ready, please submit the updated manuscript and a point-by-point response. This will help us move to a swift decision.

Handling Editor comments to the author: 

I encourage the authors to engage fully with the points raised by the reviewers. 

Reviewer Comments:

Reviewer 1

This study uses the 2024 Uganda National Population and Housing Census to analyze the prevalence and sociodemographic disparities of probable mental health disorders among people aged 10 years and older. The research topic is highly relevant to global mental health in low- and middle-income countries, the data are nationally representative and authoritative, the study design is appropriate, and the conclusions are clear. The manuscript is generally well-written. Addressing the comments below will strengthen methodological transparency, interpretability, consistency, and policy utility.

Suggestions for Revision:

1.The manuscript uses inconsistent terminology, alternating between “probable mental health disorders” and “probable conditions,” which may confuse readers about the clinical status of the identified cases. To ensure conceptual precision, consistency with WHO and mhGAP terminology, and clear differentiation between screening-based indicators and formal clinical diagnoses, I recommend using the term “probable mental health conditions” uniformly throughout the manuscript.

2.The manuscript states that mental health outcomes were “based on self reported symptoms synthesised by the Uganda Bureau of Statistics into six indicators.” However, specific instruments, item wording, coding, scoring algorithms, and thresholds for defining each “probable” condition are not provided. Without this detail, readers cannot evaluate validity, comparability with standardized tools (e.g., PHQ 9, GAD 7), or misclassification bias. Please add a dedicated subsection under Methods (e.g., “Measurement of Mental Health Indicators”) that clearly reports the exact census questions used for each symptom, response options (binary yes/no, Likert scale, etc.), how UBOS aggregated responses into the six probable mental health indicators, thresholds or criteria used to define caseness for each indicator with justification, and a reference to official UBOS technical documentation if available.

3.The study population includes individuals aged 10 years and above, but adolescents aged 10–18 years have unique developmental, cognitive, and cultural characteristics. The manuscript does not justify whether the same symptom questions are valid, developmentally appropriate, or interpretable for adolescents compared with adults. Please explicitly address whether the measurement items were validated or piloted for adolescents; if not, clearly discuss this limitation in the Methods or Limitations section.

4.The term “burden” is used repeatedly throughout the manuscript but is not appropriately justified. Disease burden represents a comprehensive summary measure of population health loss, including mortality, disability, quality of life, and socioeconomic impact, often quantified using metrics such as DALYs. This study only reports prevalence rates derived from self reported symptoms, with no data on severity, functional impairment, mortality, or economic consequences. Please replace or qualify “burden” in the title, abstract, and main text with more accurate and precise terms such as prevalence, caseload, frequency, population level symptoms, or reported distress.

5.The description of data analysis procedures is overly brief and lacks key transparency details. Please explicitly explain how prevalence rates were calculated, including clear definitions of numerators and denominators and whether percentages are computed for the total population or within specific subgroups. Please also report the proportion of missing data for mental health indicators and how missing values were handled during analysis.

6.The study covers individuals aged 10 years and older, encompassing adolescents, young adults, middle aged adults, and older adults—groups with distinctly different mental health risks and symptom patterns. Age is a well established critical determinant of mental health prevalence and expression, yet the manuscript provides no age stratified prevalence estimates and does not discuss age related disparities. Please clarify whether age stratified analyses were conducted. If such results are not included, explain the rationale and acknowledge this gap as an important limitation.

7.While the observed sub regional variation in general psychological distress is striking and policy relevant, the manuscript currently attributes this variation directly to regional differences in underlying prevalence without acknowledging the risk of ecological fallacy. Geographic differences may reflect heterogeneous case detection, reporting biases, cultural variation in expressing distress, or unmeasured contextual confounders rather than true differences in population prevalence. In the Discussion section, please explicitly acknowledge this limitation.

Thank you for the opportunity to review this important contribution to global mental health. 

Reviewer 2

Comments

Abstract

Major Comments

1. Could you clarify more explicitly that the outcomes are based on census-derived symptom indicators rather than clinically diagnosed mental disorders?

2. The phrase “probable mental health disorders” may be too strong given the measurement approach. Could this be reworded to better reflect symptom-based estimates?

3. The conclusion may sound more definitive than the data allow. Could you soften it slightly to reflect the descriptive nature of the study?

Minor Comments

4. The methods could briefly indicate how the mental health variables were constructed to improve clarity for readers.

Introduction

Major Comments

1. Some statements appear quite broad. Please make them more precise and clearly tied to the Ugandan context

2. It would help to clearly distinguish between mental health symptoms, screening indicators, and diagnosed disorders early in the introduction.

Minor Comments

3. Some claims would benefit from more recent or multiple references. Could you review and update the

4. citations where needed?

Methods

Major Comments

5. Please explain more clearly how individual symptom questions were used to define categories such as probable depression, anxiety, bipolar disorder, and psychosis

6. How were responses obtained? were they self-reported, proxy-reported, or both?

7. Clarify on whether there was a defined recall period for the symptom questions?

8. The definition of general psychological distress as the presence of at least one symptom seems broad. Could you explain the rationale and implications of this definition?

Minor Comments

9. Consider briefly noting any limitations related to translation or interpretation of questions across languages.

Results

Major Comments

10. Please ensure that all reported percentages are fully consistent between the text, tables, and figures.

11. Some descriptions of regional variation feel selective. Could you present the pattern more systematically across all regions?

12. Since the study includes individuals aged 10 years and above, could you include age-disaggregated findings or explain why they are not available?

Minor Comments

13. Some wording in this section sounds slightly interpretive. It may help to keep the language strictly descriptive.

Discussion

Major Comments

14. Some interpretations go beyond what the data can support. Consider revising the language to be more cautious throughout

15. Where differences by sex, region, or residence are discussed, could you present these as possible explanations rather than conclusions?

16. The explanation of regional differences is interesting but sometimes speculative. Could you focus more on evidence-based interpretations?

17. The suggestion linking lower burden to the presence of universities or referral hospitals may need to be reconsidered or better justified.

Minor Comments

18. When using terms such as “higher burden” or “longer,” could you define or contextualize them more clearly?

19. You may consider adding a short paragraph explaining the value of census data for mental health surveillance despite measurement limitations.

Limitations

Major Comments

20. The limitations section could be expanded. Could you discuss more explicitly the challenges of using census-based mental health indicators?

21. Consider addressing potential issues such as proxy reporting, stigma, and underreporting of symptoms?

Minor Comments

22. It would be helpful to clearly state that these findings should not be interpreted as clinical prevalence estimates.

Conclusion

Major Comments

23. The conclusions could be more closely aligned with the data presented. Avoid overly general statements

24. The recommendations are somewhat broad. It would be helpful to make them more specific and directly linked to your findings

Minor Comments

25. It would strengthen the paper to include clearer future research directions, such as qualitative or longitudinal studies.

Language and Referencing

26. There are several typographical and grammatical errors that should be corrected before publication.

27. Please check the reference list for duplicate entries and ensure consistent formatting.

28. It would also help to use terminology consistently throughout the manuscript when referring to symptoms versus disorders.

Sincerely,

Dr. Limkile Mpofu

Cambridge Prisms: Global Mental Health

# Editor report: Decision: Prevalence and regional disparities in probable mental health conditions among persons aged 10 years and older in Uganda: Evidence from the 2024 National Population and Housing Census — R0/PR5

**DOI:** 10.1017/gmh.2026.10237.pr5

**Affiliations:** London School of Hygiene & Tropical Medicine, United Kingdom of Great Britain and Northern Ireland

No accompanying comment.

# Reviewer report: Review: Prevalence and regional disparities in probable mental health conditions among persons aged 10 years and older in Uganda: Evidence from the 2024 National Population and Housing Census — R1/PR7

**DOI:** 10.1017/gmh.2026.10237.pr7

The authors have thoroughly addressed most prior comments and substantially improved the manuscript. Methodological transparency, terminology consistency, and interpretative caution have been notably strengthened. Minor revisions are required before final acceptance.

1. Throughout the manuscript, bipolar affective disorder/bipolar affective condition should be updated to bipolar disorder (bipolar condition)—the currently standard and preferred term in clinical and epidemiological research.

2.In Tables 3, 4 and 5, change the column heading from “Disorder” to “Condition” to maintain full terminological consistency.

The manuscript is scientifically sound, policy‑relevant, and nearly ready for publication. Completing these two minor revisions will finalize the paper. Thank you for the opportunity to review this valuable work.

# Editor report: Recommendation: Prevalence and regional disparities in probable mental health conditions among persons aged 10 years and older in Uganda: Evidence from the 2024 National Population and Housing Census — R1/PR8

**DOI:** 10.1017/gmh.2026.10237.pr8

**Affiliations:** Psychology, UNISA, South Africa

Dear Dr. Ainamani,

Thank you for thoroughly addressing most of the prior comments by the reviewers. This has substantially improved the manuscript. Methodological transparency, terminology consistency, and interpretative caution have been notably strengthened. 

Minor revisions are required before final acceptance of your manuscript.

Address the following please.

1. Throughout the manuscript, bipolar affective disorder/bipolar affective condition should be updated to bipolar disorder (bipolar condition)—the currently standard and preferred term in clinical and epidemiological research.

2.In Tables 3, 4 and 5, change the column heading from “Disorder” to “Condition” to maintain full terminological consistency.

The reviewers agree that the manuscript is scientifically sound, policy‑relevant, and nearly ready for publication. Completing these two minor revisions will finalize your paper. 

Thank you for this valuable work you have submitted to our Journal. 

Sincerely,

Dr. Limkile Mpofu (Handling Editor)

Cambridge Prisms: Global Mental Health

# Editor report: Decision: Prevalence and regional disparities in probable mental health conditions among persons aged 10 years and older in Uganda: Evidence from the 2024 National Population and Housing Census — R1/PR9

**DOI:** 10.1017/gmh.2026.10237.pr9

**Affiliations:** London School of Hygiene & Tropical Medicine, United Kingdom of Great Britain and Northern Ireland

No accompanying comment.

# Editor report: Recommendation: Prevalence and regional disparities in probable mental health conditions among persons aged 10 years and older in Uganda: Evidence from the 2024 National Population and Housing Census — R2/PR11

**DOI:** 10.1017/gmh.2026.10237.pr11

**Affiliations:** Psychology, UNISA, South Africa

Dear Dr. Ainamani,

We are pleased to confirm that your Manuscript ID: GMH-2026-0079, Manuscript Title: “Prevalence and regional disparities in probable mental health conditions among persons aged 10 years and older in Uganda: Evidence from the 2024 National Population and Housing Census”, has received an acceptance decision from our Editorial Team. Congratulations on this significant achievement! We truly appreciate your hard work and dedication in ensuring that your research is scientifically sound and of high quality.

We will work closely with the corresponding author (Dr. Ainamani) to complete the final steps toward publication. 

Sincerely,

Dr. Limkile Mpofu

Cambridge Prisms: Global Mental Health

# Editor report: Decision: Prevalence and regional disparities in probable mental health conditions among persons aged 10 years and older in Uganda: Evidence from the 2024 National Population and Housing Census — R2/PR12

**DOI:** 10.1017/gmh.2026.10237.pr12

**Affiliations:** London School of Hygiene & Tropical Medicine, United Kingdom of Great Britain and Northern Ireland

No accompanying comment.
